# Mesenchymal stem cells derived from perinatal tissues for treatment of critically ill COVID-19-induced ARDS patients: a case series

**DOI:** 10.1186/s13287-021-02165-4

**Published:** 2021-01-29

**Authors:** Seyed-Mohammad Reza Hashemian, Rasoul Aliannejad, Morteza Zarrabi, Masoud Soleimani, Massoud Vosough, Seyedeh-Esmat Hosseini, Hamed Hossieni, Saeid Heidari Keshel, Zeinab Naderpour, Ensiyeh Hajizadeh-Saffar, Elham Shajareh, Hamidreza Jamaati, Mina Soufi-Zomorrod, Naghmeh Khavandgar, Hediyeh Alemi, Aliasghar Karimi, Neda Pak, Negin Hossieni Rouzbahani, Masoumeh Nouri, Majid Sorouri, Ladan Kashani, Hoda Madani, Nasser Aghdami, Mohammad Vasei, Hossein Baharvand

**Affiliations:** 1 Chronic Respiratory Diseases Research Center (CRDRC), National Research Institute of Tuberculosis and Lung Diseases (NRITLD), Shahid Beheshti University of Medical Sciences, Tehran, Iran; 2 Thoracic Research Center, Tehran University of Medical Sciences, Tehran, Iran; 3 Pulmonary Department, Shariati Hospital, Tehran University of Medical Sciences, Tehran, Iran; 4 Department of Regenerative Medicine, Cell Science Research Center, Royan Institute for Stem Cell Biology and Technology, ACECR, Tehran, Iran; 5 Hematology and Cell Therapy Department, Faculty of Medical Sciences, Tarbiat Modares University, Tehran, Iran; 6 Department of Tissue Engineering and Applied Cell Science, School of Advanced Technologies in Medicine, Shahid Beheshti University of Medical Sciences, Tehran, Iran; 7 Student Research Committee, School of Nursing and Midwifery, Shahid Beheshti University of Medical Sciences, Tehran, Iran; 8 Center for Research and Training in Skin Diseases and Leprosy (CRTSDL), Tehran University of Medical Sciences, Tehran, Iran; 9 Department of Diabetes, Obesity, and Metabolism, Cell Science Research Center, Royan Institute for Stem Cell Biology and Technology, ACECR, Tehran, Iran; 10 Advanced Therapy Medicinal Product Technology Development Center (ATMP-TDC), Cell Science Research Center, Royan Institute for Stem Cell Biology and Technology, ACECR, Tehran, Iran; 11 Department of Pathology, Shariati Hospital, Tehran University of Medical Sciences, Tehran, Iran; 12 Non-Commuting Diseases Research Center (NCDRC), Fasa University of Medical Sciences, Fasa, Iran; 13 Department of Radiology, Children’s Medical Center, Tehran University of Medical Sciences, Tehran, Iran; 14 Department of Medical Immunology, Faculty of Medicine, Aja University of Medical Sciences, Tehran, Iran; 15 Iranian Research Center for HIV/AIDS, Iranian Institute for Reduction of High-Risk Behaviors, Tehran University of Medical Sciences, Tehran, Iran; 16 Digestive Disease Research Institute, Tehran University of Medical Sciences, Tehran, Iran; 17 Department of Obstetrics and Gynecology, Arash Hospital, Tehran University of Medical Sciences, Tehran, Iran; 18 Cell-based Therapies Research Center, Digestive Disease Research Institute, Shariati Hospital, Tehran University of Medical Science, Tehran, Iran; 19 Department of Stem Cells and Developmental Biology, Cell Science Research Center, Royan Institute for Stem Cell Biology and Technology, ACECR, Tehran, Iran; 20 Department of Cell and Developmental Biology, Faculty of Sciences and Advanced Technologies in Biology, University of Science and Culture, Tehran, Iran

**Keywords:** COVID-19, SARS-CoV-2, Pneumonia, Acute respiratory distress syndrome, Mesenchymal stromal cells, Cell therapy, Placenta, Umbilical cord

## Abstract

**Background:**

Acute respiratory distress syndrome (ARDS) is a fatal complication of coronavirus disease 2019 (COVID-19). There are a few reports of allogeneic human mesenchymal stem cells (MSCs) as a potential treatment for ARDS. In this phase 1 clinical trial, we present the safety, feasibility, and tolerability of the multiple infusions of high dose MSCs, which originated from the placenta and umbilical cord, in critically ill COVID-19-induced ARDS patients.

**Methods:**

A total of 11 patients diagnosed with COVID-19-induced ARDS who were admitted to the intensive care units (ICUs) of two hospitals enrolled in this study. The patients were critically ill with severe hypoxemia and required mechanical ventilation. The patients received three intravenous infusions (200 × 10^6^ cells) every other day for a total of 600 × 10^6^ human umbilical cord MSCs (UC-MSCs; 6 cases) or placental MSCs (PL-MSCs; 5 cases).

**Findings:**

There were eight men and three women who were 42 to 66 years of age. Of these, six (55%) patients had comorbidities of diabetes, hypertension, chronic lymphocytic leukemia (CLL), and cardiomyopathy (CMP). There were no serious adverse events reported 24–48 h after the cell infusions. We observed reduced dyspnea and increased SpO2 within 48–96 h after the first infusion in seven patients. Of these seven patients, five were discharged from the ICU within 2–7 days (average: 4 days), one patient who had signs of acute renal and hepatic failure was discharged from the ICU on day 18, and the last patient suddenly developed cardiac arrest on day 7 of the cell infusion. Significant reductions in serum levels of tumor necrosis factor-alpha (TNF-α; *P* < 0.01), IL-8 (*P* < 0.05), and C-reactive protein (CRP) (*P* < 0.01) were seen in all six survivors. IL-6 levels decreased in five (*P* = 0.06) patients and interferon gamma (IFN-γ) levels decreased in four (*P* = 0.14) patients. Four patients who had signs of multi-organ failure or sepsis died in 5–19 days (average: 10 days) after the first MSC infusion. A low percentage of lymphocytes (< 10%) and leukocytosis were associated with poor outcome (*P* = 0.02). All six survivors were well with no complaints of dyspnea on day 60 post-infusion. Radiological parameters of the lung computed tomography (CT) scans showed remarkable signs of recovery.

**Interpretation:**

We suggest that multiple infusions of high dose allogeneic prenatal MSCs are safe and can rapidly improve respiratory distress and reduce inflammatory biomarkers in some critically ill COVID-19-induced ARDS cases. Patients that develop sepsis or multi-organ failure may not be good candidates for stem cell therapy. Large randomized multicenter clinical trials are needed to discern the exact therapeutic potentials of MSC in COVID-19-induced ARDS.

**Supplementary Information:**

The online version contains supplementary material available at 10.1186/s13287-021-02165-4.

## Introduction

Coronavirus disease 2019 (COVID-19) is a pandemic disease caused by a new coronavirus called severe acute respiratory syndrome coronavirus 2 (SARS-CoV-2). This virus was first reported in Wuhan, China, in December 2019 [1, 2]. COVID-19 has a broad spectrum of clinical respiratory along with non-respiratory presentations that include a mild or severe flu-like syndrome, pneumonia, or respiratory failure and may end in sepsis with multi-organ failure. The most common cause of admission to the intensive care unit (ICU) is respiratory failure due to acute respiratory distress syndrome (ARDS) [3–5]. ARDS is a devastating lung injury during an uncontrolled inflammatory process that causes severe alveolar damage and capillary basement membrane leakage, which leads to progressive respiratory failure. To date, there is no effective treatment for ARDS, and a wide range of treatments has been suggested, including cell-based therapies [6, 7].

Successful repair and regeneration of endothelial and alveolar cells [8] and modulation of excessive inflammatory immune responses could be the key steps for recovery of ARDS in affected patients. Mesenchymal stem cells (MSCs) are non-hematopoietic cells that have a high proliferative ability with multi-lineage differentiation capabilities; they can be isolated from bone marrow (BM), adipose tissue, placental tissue, the umbilical cord, and other tissues [9]. MSCs have high regenerative capacities and augment tissue repair. These cells have the capability to modulate the inflammatory immune response, enhance pathogen clearance, and reduce the severity of injuries in some preclinical [10] and clinical studies. In addition, due to the lack of expression of MHC Class II on their surface, they have low immunogenicity, which favors their usage for allogeneic transplantation [11, 12].

Intravenous delivery of MSCs enables the majority of these cells to become trapped in the lung’s capillary beds within a few minutes [13–15]. The intravenous route for transplanted MSCs can effectively deliver high number of these cells to the lungs [16], which are the primary affected organ in ARDS. These potential benefits of MSCs make them candidates for a potential new treatment in patients with ARDS [8]. Since 2014, there are some clinical trials which have used MSCs obtained from variable sources (BM, fat, umbilical cord, and menstrual blood) to treat ARDS. Few of these clinical trials are ongoing and a few have announced their final reports [17–19]. At the time of writing this manuscript, there are more than 30 MSC-based clinical trials for COVID-19 registered at the World Health Organization-International Clinical Trial Registry Platform (WHO-ICTRP) and at the NIH ClinicalTrials.gov website [17]. Thus far, two case series from China and Spain have been published that addressed the safety and effectiveness of MSCs in COVID-19-induced ARDS [18, 19]. Leng and colleagues reported significant improvements in outcomes of all seven COVID-19 pneumonia patients who received 1 × 10^6^ cells/kg of commercially supplied MSCs [20]. And, Sánchez-Guijo et al. presented the results of intravenous administration of adipose tissue-derived MSCs in 13 severe COVID-19 pneumonia cases under mechanical ventilation and observed the patients 16 days after the infusions [18]. The source of the stem cells in the first study was not declared and the researchers in the second study used adipose tissue stem cells.

In this study, we aimed to assess the safety, feasibility, and tolerability of MSCs derived from human perinatal tissues (placenta and umbilical cord) in patients diagnosed with COVID-19-induced ARDS. Advantages of MSCs from perinatal sources compared to adult sources include easily available, lack of donor site morbidity, cell naivety, abundance of stem cells in the primary tissue, and high capacity for proliferation [21]. This is a 60-day follow-up report of a phase 1, two-center, open-label, single-arm trial conducted in critically ill patients diagnosed with COVID-19-induced ARDS.

## Materials and methods

### Patient eligibility

Critically ill adult patients who had hypoxemic respiratory failure and were admitted to the ICU of two hospitals were enrolled to this study. Using the WHO guideline for definition and classification of ARDS [22], patients with SpO2/FiO2 ≤ 315, SOFA score between 2 and 13 point, required mechanical ventilation (invasive or non-invasive), with SARS-CoV-2 pneumonia confirmation by either RT-PCR or chest X-ray, were considered as eligible patients for cell therapy [22]. The criteria for patient recruitment are shown in Table 1. This is a descriptive report of patients recruited from March 15, 2020, to April 10, 2020. The survivors were followed for 60 days after cell infusions.

**Table 1:** Inclusion and exclusion criteria

Inclusion criteria	Exclusion criteria
• Available informed consent • Male or female, 18–70 years of age • Evidence of pneumonia by chest X-ray or CT scan and/or confirmation of SARS-CoV-2 by qRT-PCR • ARDS diagnosed and SpO2/FiO2 ≤ 315 • SOFA score between 2 and 13 • Required mechanical ventilation and/or supplemental oxygen	• Presence of severe allergic reaction after stem cell infusion • Psychosis or under treatment for malignancy • Co-infection with HIV, tuberculosis, adenovirus, or other respiratory infections virus • Patient with previous history of pulmonary embolism • Anticipated death within 48 h • Continuous use of immunosuppressive agents or organ transplant within the past 6 months • Pregnancy or breastfeeding

*ARDS* acute respiratory distress syndrome, *SARS-CoV-2* severe acute respiratory syndrome coronavirus 2, *CT* computed tomography, *SOFA* Sequential Organ Failure Assessment

### Mesenchymal stem cell (MSC) preparation and infusion

We used allogeneic clinical-grade human prenatal MSCs that originated from either the umbilical cord (UC-MSC) or placenta (PL-MSC) tissues. The cells were evaluated for sterility, presence of mycoplasma, and endotoxin levels. The trypan blue exclusion method was used to evaluate cell viability.

UC-MSCs were derived from umbilical cord tissues of informed healthy donors who provided consent for the use of their tissues. Briefly, the umbilical cords were rinsed in PBS, cut into 2–3 mm pieces, and digested by enzyme cocktails. These cells were subsequently cultivated, passaged, and harvested at passage-4. The harvested cells were characterized by flow cytometry (Supplementary Fig. 1), frozen, and stored until use. The cryopreserved UC-MSCs were thawed and washed to remove dimethyl sulfoxide and subsequently suspended in 100 ml normal saline with 5% w/w human serum albumin for each infusion. The placental MSCs (PL-MSCs) were prepared from fresh placental tissue as previously reported [23] and administered fresh. The PL-MSCs were suspended in 100 ml of normal saline supplemented with 2% w/w human serum albumin for each infusion. The total number of UC-MSCs (thawed) or PL-MSCs (fresh) was calculated to be 200 × 10^6^ cells per infusion.

Six patients received freeze/thawed UC-MSCs and 5 received fresh PL-MSCs. Each patient received a total dose of 600 × 10^6^ allogeneic human MSCs by intravenous infusions that were divided into three doses administered every other day. The infusion time was approximately 30–45 min at a speed of approximately 50 drops/min. All patients received standard medications according to their individual conditions.

### Outcome measurement

The main outcome of the study was to assess the safety and potential adverse events following transplantation of repeated doses of perinatal tissue MSCs in COVID-19-induced ARDS patients. Potential safety concerns for MSC infusion administered over a brief period of time (24–48 h). Early adverse events were defined as follows: allergic reactions that typically comprise maculopapular rashes and/or urticaria without fever or hypotension; anaphylactic reactions that manifest as worsening of dyspnea, wheezing, anxiety, hypotension without fever, and bronchospasms in severe cases; and cell embolization in the lungs or less commonly in the heart caused by large aggregations of cells during the IV infusion that result in deteriorated organ function [24]. In case of any severe anaphylactic reaction or embolization, the study would be terminated. Patients were followed for 60 days post-treatment. We also evaluated improvement in SpO2 after infusion and mortality rate in the treated cases.

### Statistical analysis

Descriptive data are presented as the mean (standard deviation [SD]), median (interquartile range [IQR]), and number (%). The two-sample *t* test and chi-square test were used to assess the differences between survivors and non-survivors. The paired *t* test was used to compare the variables before and after intervention. The tests were two-sided and a *P* value of < 0.05 was considered statistically significant.

## Results

### Patients

There were 11 patients (8 males and 3 females) with a mean age of 53.8 (SD 10.37) years who were recruited for this study. At the time of admission, all patients were dyspneic and had respiratory rates of more than 30 breaths per minute. All needed oxygen supplementation with FiO2 that was more than 40%, their SpO2 levels at room air were less than 86%, and the SpO2/FiO2 was less than 315. At the time of the infusion, nine cases required noninvasive respiratory support, two had been intubated for 2 days, and one was under extracorporeal membrane oxygenation (ECMO) therapy. From the 11 enrolled patients, six had the following comorbidities: hypertension (*n* = 1), diabetes mellitus (*n* = 1), diabetes mellitus and cardiomyopathy (CMP) (*n* = 1), diabetes mellitus and hypertension (*n* = 2), and chronic lymphocytic leukemia (CLL) (*n* = 1). With the exception of case no #8 from the survivors, the remaining patients’ laboratory data were within normal limits from the time of admission until the time of the cell infusions. Tables 2 and 3 show the basic demographic variables, treatments, and average baseline characteristics of the surviving and non-surviving critically ill patients with COVID-19 at the time of the cell infusions, respectively.

**Table 2:** Patients’ basic demographic variables and treatments

	Survivors						Non-survivors				
**Patient ID**	2	3	4	8	10	11	1*	5	6	7	9
**Hospital**	1	1	2	1	2	2	1	2	2	1	2
Cell type**	UC-MSC	UC-MSC	UC-MSC	PL-MSC	PL-MSC	PL-MSC	UC-MSC	UC-MSC	UC-MSC	PL-MSC	PL-MSC
Age (years)***	60–65	55–60	65–70	60–65	40–45	45–50	65–70	50–55	55–60	35–40	60–65
Sex (1/2)***	2	2	1	1	1	1	2	1	1	1	1
**Weight (kg)**	80	69	60	95	85	103	80	80	87	85	94
**Underlying disease**	HTN, DM	–	CLL	CMP, DM	–	HTN	HTN, DM	–	–	–	DM
**Treatment protocol**	HCQ, Kaletra	HCQ, Kaletra	HCQ, Kaletra, Azithromycin, IVIG, Ribavarin	HCQ, Kaletra	Meropenem, Vancomycin, IVIG, Favipiravir	Kaletra, Meropenem, Vancomycin, Tamiflu, IVIG, Favipiravir	HCQ, Kaletra	HCQ, Kaletra, Azithromycin, IVIG, + ECMO	Kaletra, Ribavirin, Vancomycin, Imiper, IVIG	HCQ, Kaletra	Kaletra, Meropenem, Colistin, IVIG
**qRT-PCR**	+	+	+	+	+	+	−	+	+	+	+

*HCQ* hydroxychloroquine, *HTN* hypertension, *DM* diabetes mellitus, *IVIG* intravenous immunoglobulin, *ECMO* extracorporeal membrane oxygenation, *qRT-PCR* quantitative reverse transcription-polymerase chain reaction, *CMP* cardiomyopathy, *CLL* chronic lymphocytic leukemia, *UC-MSC* umbilical cord mesenchymal stem cells, *PL-MSC* placental MSC

*qRT-PCR in this patient was initially negative but history, clinical presentation, and radiography of the patient were indicative of COVID-19 infection

**UC-MSCs were freeze/thawed prior to infusion; however, PL-MSCs were freshly administered

***To avoid the patients/participants identity being revealed, age presented as a range and gender as (1/2)

**Table 3:** Average baseline characteristics in 11 patients with COVID-19

	Survivors (***n*** = 6)	Non-survivors (***n*** = 5)	All (***n*** = 11)
Age (years)*	53.50 ± 10.50	54.20 ± 11.50	53.80 ± 10.40
**Sex**			
**Male**	4 (80%)	4 (66.70%)	8 (73%)
**Female**	1 (20%)	2 (33.30%)	3 (27%)
**Comorbid disease**	4	2	6 (54.50%)
**Fever (> 37.3 °C)**	6 (100%)	4 (80%)	10 (91%)
**Cough**	5 (83.30%)	5 (100%)	10 (91%)
**Dyspnea**	5 (83.30%)	5 (100%)	10 (91%)
**PaO2/FiO2 ratio (IQR)**	87.67 (15.00)	69.40 (47.50)	79.39 (35)
**Respiratory rate (> 30)**	6 (100%)	5 (100%)	11 (100%)
**SOFA (min-max)**	3–6	3–7	3–7
**WBCs**	6668 (1451)	14,957 (6581)	10,352 (6160)
**Lymphocytes (%)**	21.18 (18.00)	5.56 (5.10)	14.08 (8.70)
**Lymphocytes < 1000**	5 (83%)	4 (80%)	9 (81.8%)
**Platelets**	199,600 (58439)	178,500 (58563)	190,223 (55831)
**PT**	15.64 (4.72)	14.50 (0.55)	15.13 (3.41)
**PTT**	32.60 (11.59)	38.25 (9.95)	35.11 (10.64)
**BUN**	22.88 (11.26)	35.95 (23.18)	28.69 (17.67)
**Cr**	1.04 (0.29)	1.76 (1.17)	1.36 (0.83)
**LDH**	1237 (209)	1122 (500)	1186.33 (345.64)
**Median days from first symptom to admission (IQR)**	4.5 (10)	11 (4)	10 (10)
**Median days from first symptom to first infusion (IQR)**	9 (16)	17 (3)	16 (10)

*Except age which is presented as mean ± SD, all data are presented as *n* (%), or median ± (IQR)

*IQR* interquartile range, *Cr* creatinine, *SOFA* Sequential Organ Failure Assessment

### Clinical course following cell transplantation

The viability of the infused MSCs ranged from 88.7 to 94.2% (mean: 92.7%). We noted that nine of the treated patients tolerated the MSC infusions and there were no acute infusion-related severe adverse events. However, two cases developed shivering that occurred during the initial PL-MSC infusion, which was relieved by supportive treatment in less than 1 h. This shivering did not develop again during the second and third infusions. None of the patients suffered from respiratory or cardiovascular complications within 36 h after the MSC infusion. Additionally, safety laboratory values [serum creatinine (Cr), bilirubin, alanine aminotransferase (ALT), aspartate aminotransferase (AST)] did not significantly increase within the following days after the cell transplantation.

Totally six patients out of eleven survived. Five patients significantly improved and were discharged from the ICU, 2 to 7 days after the infusions (Supplementary Fig. 2 and Supplementary Table 1). Most patients described significant relief of their dyspnea and there was a decrease in respiratory rate within 48–96 h after the first cell infusion. The results of the survivors showed that the median time to relief after the first infusion was 2.5 days for fever (≤ 37.2 °C), 3 days for respiratory rate (≤ 24/min), and 2 days for cough (mild or absent). The saturation of pulse oxygen significantly improved in survivors (9.2 [3.7–14.6]) compared to non-survivors (6.6 [5.01–11.0]).

One of the survivors who had comorbidities of CMP and diabetes had a significant increase in liver enzymes (ALT: 4200 U/L, AST: 11200 U/L, LDH: 7937 U/L) (Supplementary Table 2). His BUN and Cr levels had been mildly increasing for 4 days before the onset of cell infusions (data not shown). He showed a marked decrease in liver enzymes and LDH levels after the MSC infusion on day 13 after the infusion.

Despite these findings, we commenced with the cell transplantation and he showed a significant improvement in SpO2 and marked relief from dyspnea within 24–48 h. His liver enzymes decreased to half of their levels on the second infusion day; however, he developed acute renal failure on day 4. The course of his cell therapy was interrupted by frequent hemodialysis and took 12 days to complete the three doses. After the third dose, his hepatic enzymes significantly decreased (ALT: 139 U/L, AST: 122 U/L, LDH: 627 U/L) but BUN and Cr levels remained elevated. This patient remained in the ICU on intermittent nasal O2 because of pleural effusion and mild pleural edema related to his high BUN levels; however, he was discharged to the Nephrology Ward on day 18.

Five cases died 4–19 days (average: 8 days) after the first cell infusion (Supplementary Fig. 2). Two were intubated (one under ECMO therapy) and two had signs of sepsis (leukocytosis and decreased levels of consciousness). One of the patients showed signs of an increase in SpO2 and a decrease in dyspnea during three cell injections, but suddenly went into cardiac arrest on day 7 of the cell infusion. Supplementary Fig. 2 shows the hospital courses of the patients after cell therapy.

### Serum cytokine levels

**Fig. 1:**
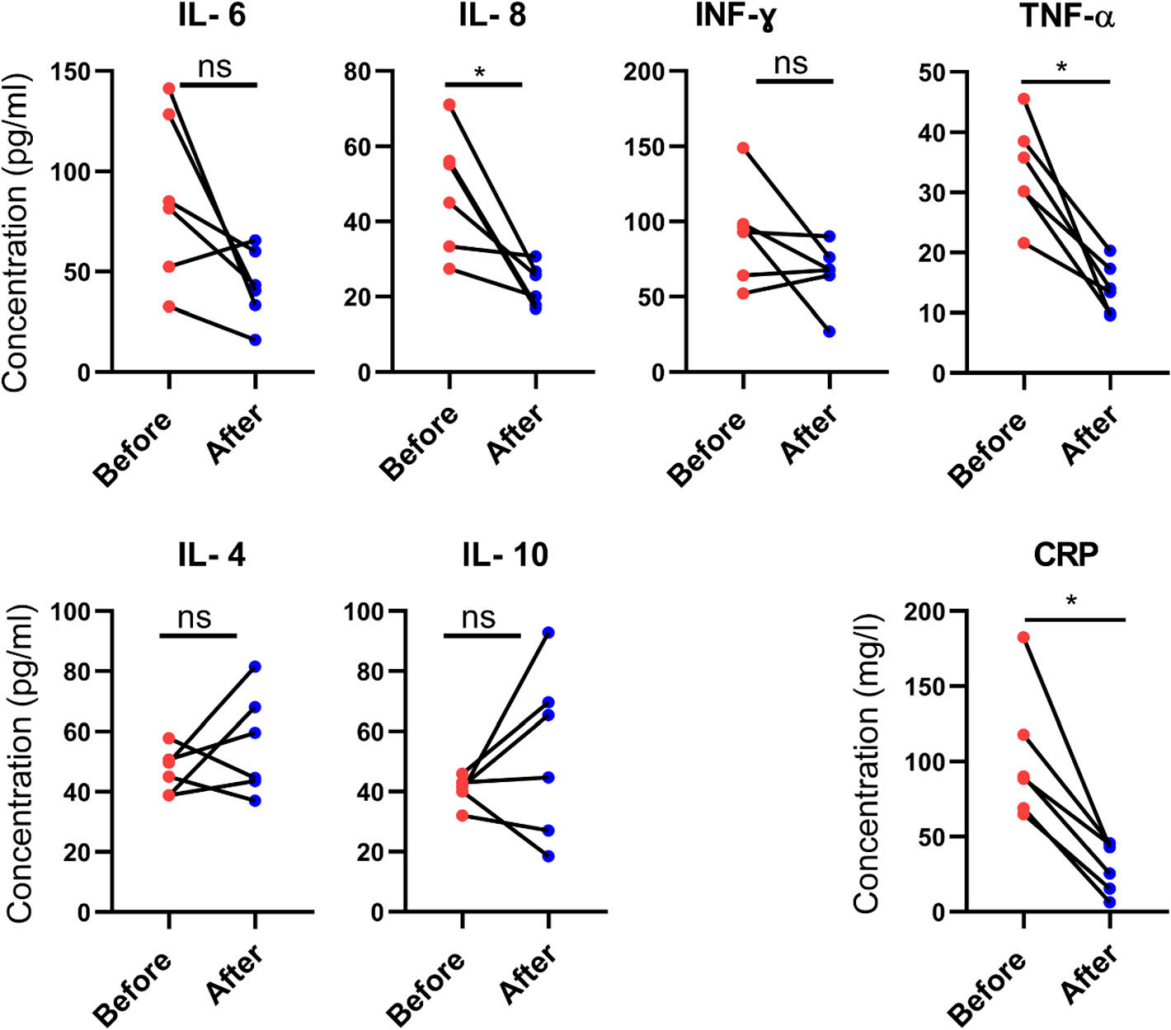
Change in patients’ serum biomarker levels on days 0 (baseline) and 5 after the first infusion. Analysis of biomarkers on before (baseline) and 5 days after the first infusion (24 h after the last infusion) demonstrated a significant reduction in IL-8, tumor necrosis factor-alpha (TNF-α), and C-reactive protein (CRP) in all six survivors. Serum IL-6 levels and interferon gamma (INF-ɣ) reduced in five and four of the recovered patients, respectively. IL-4 and IL-10 levels increased in four cases, but the differences were not statistically significant. *P < 0.05; ns, not significant

Analysis of biomarkers on days 0 (baseline) and 5 after the first infusion (24 h after the last infusion) showed a significant reduction in the pro-inflammatory biomarkers including interleukin-8 (IL-8, *P* = 0.02), tumor necrosis factor-alpha (TNF-α; *P* = 0.01), and C-reactive protein (CRP *P* = 0.01) in all six survivors. Serum IL-6 levels decreased in five (*P* = 0.06) of the recovered patients and interferon gamma (INF-ɣ) levels decreased in four (*P* = 0.14) of the recovered patients. On the other side, anti-inflammatory cytokines including IL-4 and IL-10 levels increased in four cases, but the differences were not statistically significant (*P* = 0.29) (Fig. 1).

### Lung imaging

**Fig. 2:**
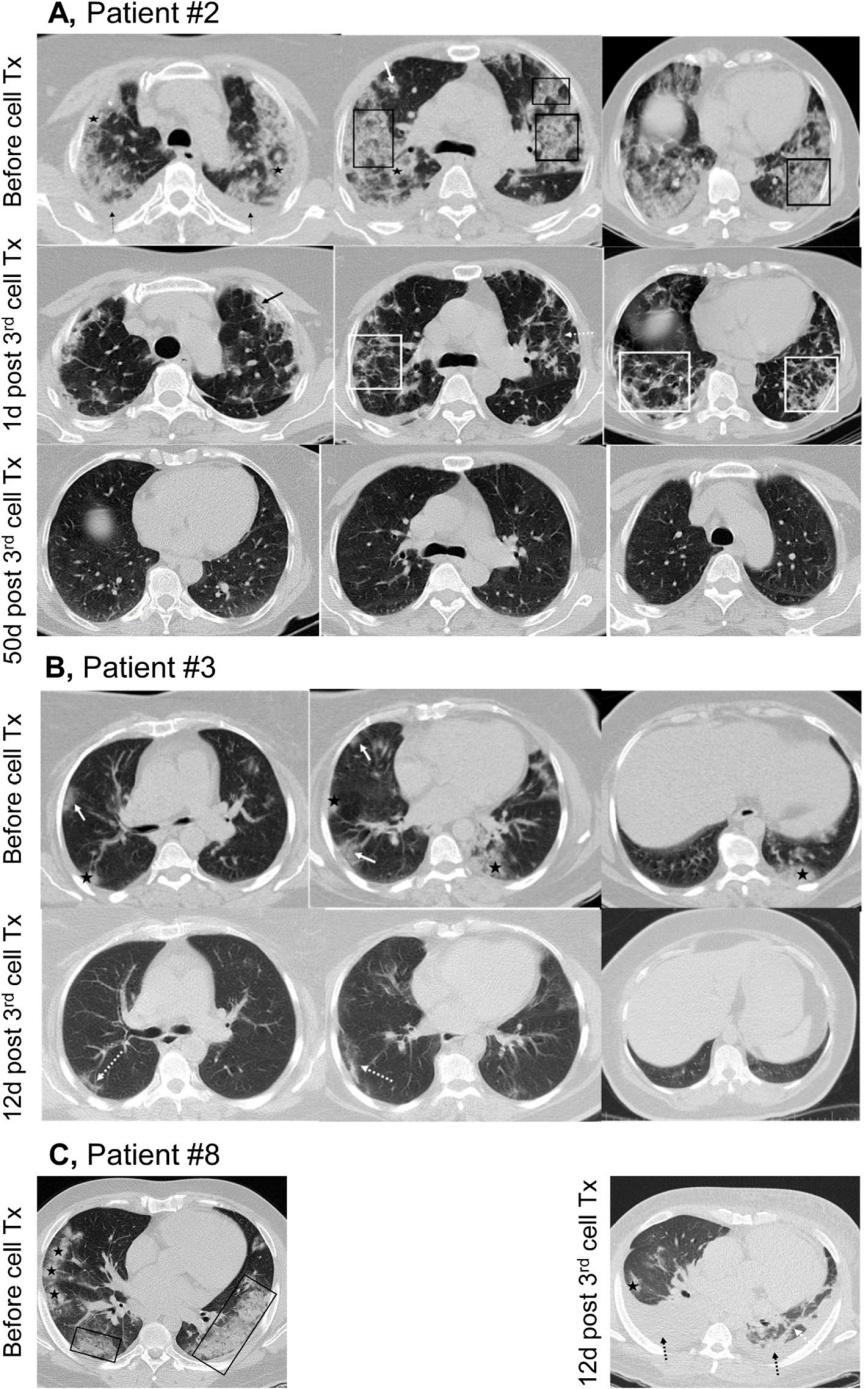
Chest computed tomography (CT) images in three survivors. **a** Patient #2: First row is prior to cell infusion. Note the extensive mixed ground glass opacities, crazy paving appearance, vascular dilatation, consolidations with peripheral subpleural dominancy, and bilateral mild pleural effusion. The CT severity score for all five lobes was 24. The second row shows CT images 1 day after the third dose of cell therapy. There is a decrease in extension of consolidations associated with band-like opacities and traction bronchiectasis are evident. CT severity score for all five lobes was 18. The third row shows near complete resolution of opacities and subpleural bands without residual fibrosis 50 days after MSC therapy. The CT severity score for all five lobes was 2. The percentage of lung involvement in each image of the first, second, and third columns was assessed at pretreatment, 1 day after the third post-cell therapy, and after 50 days of treatment, respectively, as follows. First column: about 60%, 25%, and 0%; second column: about 75%, 30%, and 0%; and third column: about 90%, 50%, and 2%. **b** Patient #3: First row shows chest CT images before cell therapy. Note the patchy areas of ground glass opacity and consolidations in the subpleural regions of the lungs. The CT severity score for all five lobes was 16. The second row shows a significant reduction in the extension of lung involvement 12 days after the third dose of cell therapy. Most consolidations had resolved completely with only band-like opacities and mild tiny residual ground glass opacities present. The CT severity score for all five lobes was 8. The percentage of lung involvement in single images from the first, second, and third columns was assessed at pretreatment and 12 days after the third cell therapy, respectively, as follows. First column: about 15% and 5%; second column: about 35% and 15%; and third column: about 20% and 3%. **c** Patient #8: Axial CT scan images from the base of the lung before cell therapy. The left image shows peripheral subpleural consolidations and ground glass opacity. At the same level, the right image shows a significant decrease in consolidations 12 days after cell therapy; however, the patient developed bilateral pleural effusion due to acute renal failure during the course of the disease. The CT severity score for all five lobes at the initial lung CT scan was 24, which decreased to 13 at 12 days after cell therapy. The percentage of lung involvement in the pretreatment image was about 60%, which decreased to 20% in the post-treatment image. In all images, different patterns of lung involvement have the following annotations: crazy paving appearance (black boxes), consolidation (black stars), pure ground glass opacity (solid white arrows), vascular dilatation (solid white arrows), traction bronchiectasis (solid black arrows), subpleural band (dashed white arrows), architectural distortion (white boxes), and pleural effusion (dashed black arrows)

Lung CT scans were scored by the obtaining the sum of the percentage of involvement of each five lung lobes, as follows: 1 (< 5% involvement), 2 (5–25% involvement), 3 (26–49% involvement), 4 (50–75% involvement), and 5 (> 75% involvement). The final score ranged from zero (no involvement) to 25 (maximum involvement) according to the Radiology Assistant Severity Classification. Lung CT scans performed prior to the MSC infusions showed that all cases had significant lung involvement, which included variable degrees of mixed ground glass opacities, crazy paving pattern, or consolidations with peripheral subpleural dominancy, in addition to vascular dilation, traction bronchiectasis, and pleural effusion in some cases. In three survived cases, lung CT were available after therapy. The lung CT scans of two patients showed significant resolution of opacities after completion of the MSC therapy. No opacities were visualized and the subpleural bands, which were indeterminate as fibrosis, had complete resolution. This finding indicated that these band-like opacities were not fibrosis. The third case (#8) developed acute renal failure, pulmonary edema, and bilateral plural effusion. However, after treatment of his pulmonary edema, there was a significant decrease in the extension of COVID-19 related opacities (Fig. 2).

### Predictive factors

The initial Sequential Organ Failure Assessment (SOFA) score was relatively the same; comorbid diseases were present in both survivors and non-survivors (Table 3). Although the time interval between the first symptom until hospital admission was less in survivors compared with non-survivors, this difference was not statistically significant because of the low numbers of patients (95% CI 2.72 to 11.99; *P* = 0.19). The mean duration of major symptoms that included severe dyspnea before admission in the survivor group was 7.1 ± 6.7 days, whereas it was 11.80 ± 2.05 days in non-survivors (*P* = 0.19). The median length of symptomatic days before the MSC infusion was 9 (range 4–32) days for patients who survived and 17 (range 15–19) days for the non-survivors (*P* = 0.25). The lymphocyte fractions (%) of total WBCs in all of the non-survivors were less than 10% (*P* < 0.01, Supplementary Table 2) compared to the normal value. The baseline lymphocyte percentage among survivors (21.18%) was higher than non-survivors (5.56%). In addition, the WBC counts of all survivors (except for the CLL case) was within normal limits, and the average counts were lower than in the deceased cases (95% CI 2023 to 13,262; *P* = 0.01).

### Patient follow-up

The five discharged cases were in good condition at the 60-day post-hospitalization follow-up. There were no complaints of dyspnea at rest or on exertion, tachypnea, or fever. At the time of writing this report, one case (patient #8) remained hospitalized because of renal failure but did not show respiratory symptoms related to COVID-19 on day 60 after the MSC infusions.

## Discussion

This case series study is a primary report of a clinical trial that aimed to assess the safety, feasibility, and tolerability of a high dose of prenatal MSCs administered in three infusions as a potential treatment for critically ill COVID-19 patients with ARDS. The most of recovered patients had rapid dramatic response in 48–96 h after the first MSC infusions. The surviving cases were well during the 60-day follow-up assessment. The only adverse event was transient shivering, which occurred once in two cases. Since this shivering was not associated with fever and disappeared in less than 1 h by supportive care, it was not caused by COVID-19 infection.

Since 2014 until the COVID-19 pandemic, approximately 30 registered clinical trials have used MSCs for ARDS. Most of these trials have not begun or are currently recruiting patients and lack an update status [25]. From these trials, there are only three published final reports from phase 1–2 studies that focused on safety, tolerability, and feasibility of MSCs for ARDS [26–28].

Two recent case series reports implied the safety and efficacy of allogenic MSC therapy in ARDS patients with COVID-19. Leng and colleagues have used ACE-2 negative MSCs from an undefined source and reported that all seven cases recovered [20]. All of their cases had SpO2 levels greater than 90% in room air (range 90% to 95%) in most days prior to the stem cell injection. The respiratory rates of these patients were not recorded in their report. The only critically severe case in their study had an average respiratory rate of less than 23/min before the injection with a face mask for mechanical ventilation. Sánchez-Guijo and colleagues also reported a mortality rate of 15% (2 out of 13 cases) with a median follow-up of 16 days using allogenic adipose tissue-derived MSCs [18]. In their study, nine patients showed improvements in respiration (53%), two were discharged from the ICU, and two additional patients remained stable. We noted that the mortality rate in their case series (15%) was much lower than mortality in our study (45%). Although the total numbers of the cases in both studies were low for comparison, this difference could be attributed to the following factors. The first factor might be the differences between the general conditions of the patients at baseline in the two studies. At the time of their report, they had still four cases in the ICU, two under mechanical ventilation and two under ECMO. Another factor could be related to the short follow-up (16 days) in their case series compared to the longer follow up period (60-day end-point report) in our study. Finally, it is well-known that ICU care is an important factor in the outcome of ARDS patients. ECMO was available at only one of our two hospitals and possible differences in the facilities for patient care might have influenced the outcome in their series.

We noted that the major inflammatory biomarkers (CRP, IL-6, IL-8, and TNF-α) significantly decreased after the MSC infusions. Although MSC regulation of the inflammatory response to SARS-Cov2 has been previously reported in humans [20, 29], the exact mechanism by which MSCs exert their therapeutic effects is not entirely clear. MSCs reduce secretion of pro-inflammatory cytokines (IFN-γ, IL-6, IL-8, and IL-1) from infiltrated immune cells [30]. The anti-inflammatory effects of MSCs on host tissues by their secretion of TGF-β, IL-10, IL-4, and prostaglandin E2 have been reported in preclinical models of ARDS and sepsis [30, 31]. MSCs also protect endothelial cells from inflammation and oxidative stress [32]. Moreover, intra-bronchial administration of MSCs markedly reduce lung edema and restore normal lung endothelial and epithelial permeability [33]. In addition, MSCs secrete high levels of growth factors, which may have a critical role in tissue repair [13]. MSCs can also reduce regulated cell death via secretion of keratinocyte growth factor (KGF), angiopoietin-1, hepatic growth factor (HGF) [34, 35], and reduction of TNF-α levels [32].

These paracrine signals of MSCs can be mediated by secretion of bilayer membranous extracellular vesicles (EVs) such as exosomes and microvesicles. The secreted EVs act as shuttling carriers between cells where they transfer proteins, lipids, and miRNAs to target cells, and induce changes in their phenotypes and functions [36]. IV infusion of allogeneic MSC-derived EVs in a recent phase 1 clinical trial showed a dramatic improvement in PaO2/FiO2 levels in severe COVID-19 patients [37].

In this study, we used IV administration because this route effectively delivers a high concentration of cells to the lungs as described in previous cell-based therapies in ARDS [18, 20, 26, 27]. The benefits of IV administration include safety and tolerability, effective delivery of a high concentration of cells to the lungs, systemic release of anti-inflammatory and anti-fibrotic factors, and the possibility for prescribing repetitive cell doses over a short course of cell therapy [16].

Our study had a number of limitations. Most of our patients were in critical condition; therefore, usage of cryo-banked samples was inevitable.

We did not evaluate differences between the therapeutic values of thawed and fresh MSCs [38], as well as the ideal time points for patient treatment, both of which might affect the outcome [16].

There was a difference in the duration of dyspnea before admission between survivors and non-survivors, and this delay in admission time might have affected the outcome of the patients. In addition, the emergency condition in ICUs and heavy traffic of patients did not allow us to carry out comparative lung function evaluations in the patients.

We also did not measure D-dimer, which is associated with a poor prognosis in COVID-19 patients [39]. This association was first reported in the literature on March 20, 2020, and we were not aware of this association at the time of the design and implementation of our study. The low numbers of patients and the variability in the treatment protocols made some weaknesses in interpretation of the results. In addition, the lack of a case-matched control group limited our ability to compare the ICU course and mortality of our MSC-treated cases with similar patients with COVID-19-induced ARDS. Although we observed a significant reduction in CRP and pro-inflammatory cytokines, because of the lack of these data in non-survivors, we cannot claim that these cytokines are good indicators for the response to treatment and recovery of ARDS patients.

In conclusion, our findings from this phase 1 trial suggest that intravenous administration of high dose of MSCs from a prenatal source is relatively safe, tolerable, and could rapidly improve respiratory symptoms and reduce inflammatory conditions in some critically ill COVID-19 patients. Although our results are promising, we are unable to conclude that MSCs therapy is dramatically effective and completely safe in COVID-19-induced ARDS. Large, randomized controlled trials are necessary to shed light on this gap in knowledge about the therapeutic potential of MSCs for the treatment of this disease.

## Supplementary Information

**Additional file 1: Figure S1**. Flow cytometric characterization of UC-MSCs. The cells were negative for (A) CD31 (endothelial marker), (B) CD45, (C) CD34 (hematopoietic stem cell markers), (D) CD11b (leukocyte marker), and (E) HLD-R (MHC-II). They displayed positive expression for MSCs markers; (F) CD105, (G) CD90, (H) CD73, and (I) CD29.

**Additional file 2: Figure S2.** Timeline for ICU admitted the patients treated with MSCs. (A) Survivors. (B) Non-survivors. Nine patients received three intravenous (IV) infusions. The course of cell therapy of patient number #8 was interrupted following frequent hemodialysis as a result of acute renal failure that developed on day 4. This patient took 12 days to complete the three doses. Patient number #1 was intubated and did not complete the course of his cell therapy and died on day 4. CRRT performed for both patients (#5 and 9) late during the course of disease and at least 48 h after completion of the cell infusion. ICU: Intensive care unit, ARDS: Acute respiratory distress syndrome, MSCs: Mesenchymal stem cells; ECMO: Extracorporeal membrane oxygenation, CRRT: Continuous renal replacement therapies, MOF: Multi-organ failure, #: Patient number.

**Additional file 3: Table S1**. Clinical data before the first (day one) and last (day 5) cell infusions.

**Additional file 4: Table S2**. Laboratory findings before the first and after the last cell infusions.

## Data Availability

All data generated or analyzed during this study are included in this published article.

## Abbreviations

ALT: Alanine aminotransferase
ARDS: Acute respiratory distress syndrome
AST: Aspartate aminotransferase
BM: Bone marrow
CLL: Chronic lymphocytic leukemia
CMP: Cardiomyopathy
COVID-19: Coronavirus disease 2019
Cr: Creatinine
CRP: C-reactive protein
CT: Computed tomography
ECMO: Extracorporeal membrane oxygenation
EVs: Extracellular vesicles
HGF: Hepatic growth factor
ICUs: Intensive care units
IFN-γ: Interferon gamma
KGF: Keratinocyte growth factor
MSCs: Mesenchymal stem cells
PL-MSCs: Placental MSCs
SARS-CoV-2: Severe acute respiratory syndrome coronavirus 2
SD: Standard deviation
SOFA: Sequential Organ Failure Assessment
TNF-α: Tumor necrosis factor-alpha
UC-MSCs: Umbilical cord MSCs
WHO-ICTRP: World Health Organization-International Clinical Trial Registry Platform
