# Supplementary material for: Mesenchymal stem cells derived from perinatal tissues for treatment of critically ill COVID-19-induced ARDS patients: a case series
Source: Stem Cell Res Ther. 2021 Jan 29;12:91. doi: 10.1186/s13287-021-02165-4 (PMC7844804; doi:10.1186/s13287-021-02165-4)

**Supplementary Figure 1. Flow cytometric characterization of UC-MSCs.** The cells were negative for (A) CD31 (endothelial marker), (B) CD45, (C) CD34 (hematopoietic stem cell markers), (D) CD11b (leukocyte marker), and (E) HLD-R (MHC-II). They displayed positive expression for MSCs markers; (F) CD105, (G) CD90, (H) CD73, and (I) CD29.


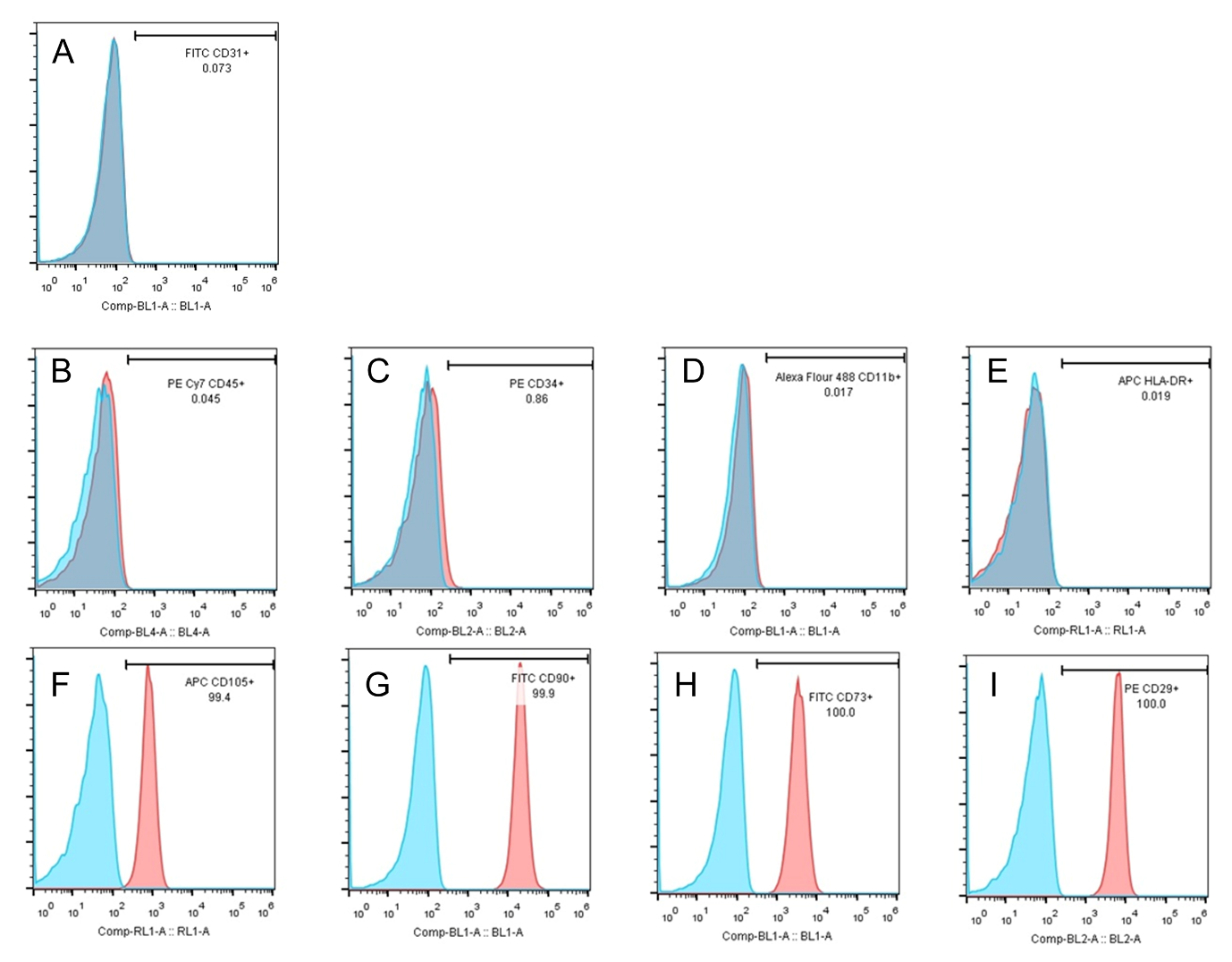

Supplement: Supplementary file 1 — Additional file 1: Figure S1. Flow cytometric characterization of UC-MSCs. The cells were negative for (A) CD31 (endothelial marker), (B) CD45, (C) CD34 (hematopoietic stem cell markers), (D) CD11b (leukocyte marker), and (E) HLD-R (MHC-II). They displayed positive expression for MSCs markers; (F) CD105, (G) CD90, (H) CD73, and (I) CD29. [file 13287_2021_2165_MOESM1_ESM.docx]
