# Supplementary material for: Mesenchymal stem cells derived from perinatal tissues for treatment of critically ill COVID-19-induced ARDS patients: a case series
Source: Stem Cell Res Ther. 2021 Jan 29;12:91. doi: 10.1186/s13287-021-02165-4 (PMC7844804; doi:10.1186/s13287-021-02165-4)

**Supplementary Figure 2. Timeline for ICU admitted the patients treated with MSCs.** (A) Survivors. (B) Non-survivors. Nine patients received three intravenous (IV) infusions. The course of cell therapy of patient number #8 was interrupted following frequent hemodialysis as a result of acute renal failure that developed on day 4. This patient took 12 days to complete the three doses. Patient number #1 was intubated and did not complete the course of his cell therapy and died on day 4. CRRT performed for both patients (#5 and 9) late during the course of disease and at least 48 hours after completion of the cell infusion. ICU: Intensive care unit, ARDS: Acute respiratory distress syndrome, MSCs: Mesenchymal stem cells; ECMO: Extracorporeal membrane oxygenation, CRRT: Continuous renal replacement therapies, MOF: Multi-organ failure, #: Patient number.


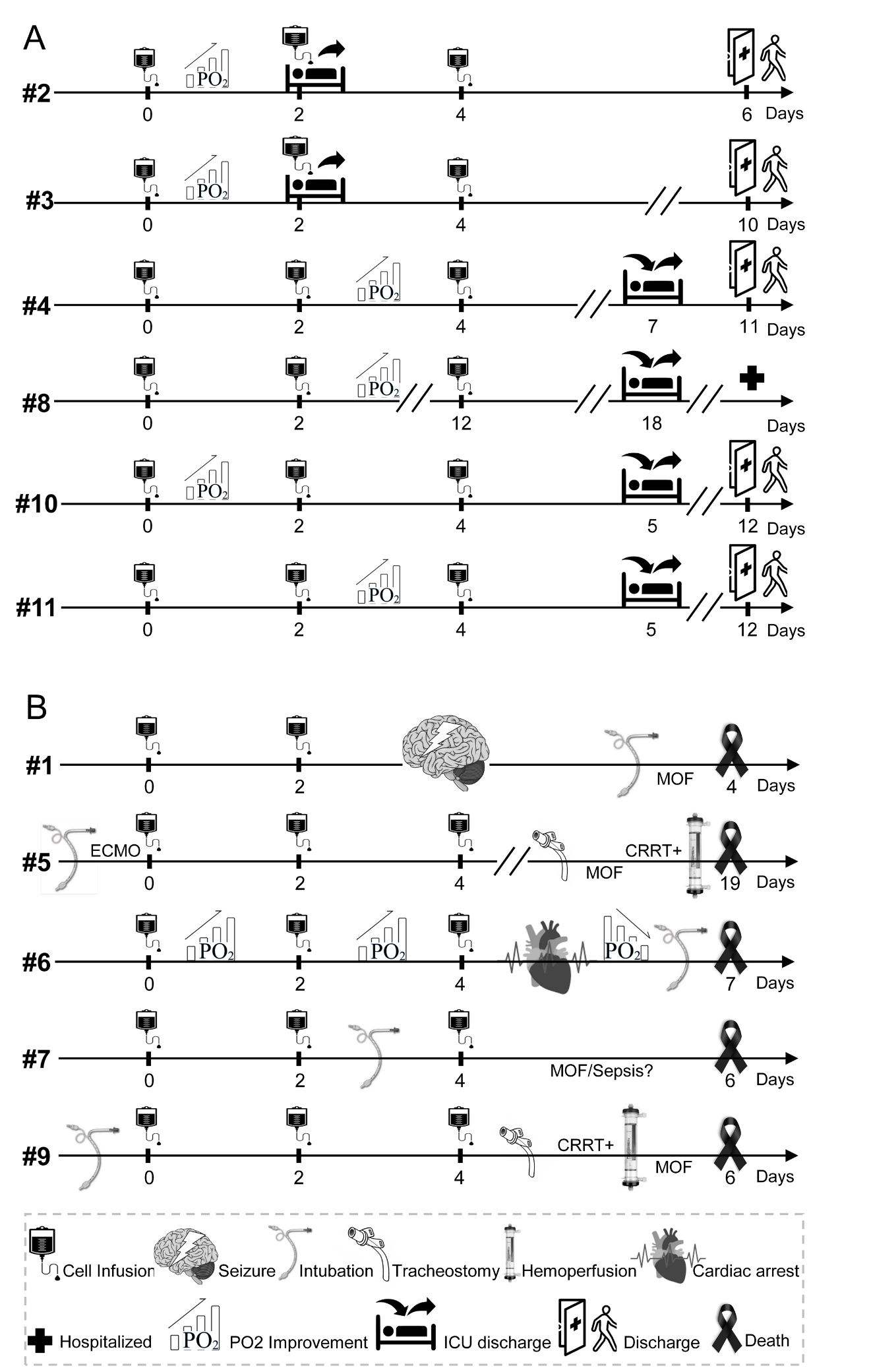

Supplement: Supplementary file 2 — Additional file 2: Figure S2. Timeline for ICU admitted the patients treated with MSCs. (A) Survivors. (B) Non-survivors. Nine patients received three intravenous (IV) infusions. The course of cell therapy of patient number #8 was interrupted following frequent hemodialysis as a result of acute renal failure that developed on day 4. This patient took 12 days to complete the three doses. Patient number #1 was intubated and did not complete the course of his cell therapy and died on day 4. CRRT performed for both patients (#5 and 9) late during the course of disease and at least 48 h after completion of the cell infusion. ICU: Intensive care unit, ARDS: Acute respiratory distress syndrome, MSCs: Mesenchymal stem cells; ECMO: Extracorporeal membrane oxygenation, CRRT: Continuous renal replacement therapies, MOF: Multi-organ failure, #: Patient number. [file 13287_2021_2165_MOESM2_ESM.docx]
