# Supplementary material for: Mesenchymal stem cells derived from perinatal tissues for treatment of critically ill COVID-19-induced ARDS patients: a case series
Source: Stem Cell Res Ther. 2021 Jan 29;12:91. doi: 10.1186/s13287-021-02165-4 (PMC7844804; doi:10.1186/s13287-021-02165-4)
Supplement: Supplementary file 3 — Additional file 3: Table S1. Clinical data before the first (day one) and last (day 5) cell infusions. [file 13287_2021_2165_MOESM3_ESM.docx]

**Supplementary Table 1. Clinical data before the first (day one) and last (day 5) cell infusions.**

|  | **Survivors** | | | | | | | | | | | | | **Non-Survivors** | | | | | | | | | | |
| --- | --- | --- | --- | --- | --- | --- | --- | --- | --- | --- | --- | --- | --- | --- | --- | --- | --- | --- | --- | --- | --- | --- | --- | --- |
| **Patient #** | 2 | | 3 | | | 4 | | 8*** | | 10 | | 11 | | 1 **** | | 5 | | | 6***** | | 7 | | 9 | |
| **Before/B After/A** | B | A | B | | A | B | A | B | A | B | A | B | A | B | A | B | | A | B | A | B | A | B | A |
| **Temp. (°C)** | 39.8 | 37 | 37.4 | | 37 | 37.6 | 37 | 39 | 37 | 37.6 | 37 | 38.7 | 37 | 37 | 37.9 | 38.8 | | 37 | 38.6 | 37.2 | 38.2 | 37 | 38.5 | 38 |
| **Cough** | Yes | No | No | | No | Yes | No | Yes | No | Yes | No | Yes | No | Yes | NA | Yes | | NA | Yes | NA | Yes | NA | Yes | NA |
| **Sputum** | No | No | No | | No | Yes | No | No | No | No | No | Yes | No | No | NA | No | | Yes | No | Yes | No | No | Yes | NA |
| **Diarrhea** | No | No | No | | No | Yes | No | No | No | No | No | No | No | No | No | No | | No | No | No | No | No | ? | ? |
| **Shortness of breath** | Yes | No | Yes | | No | Yes | No | Yes | Yes | Yes | No | Yes | No | Yes | NA | Yes | | NA | Yes | No | Yes | NA | Yes | NA |
| **Respiratory rate /min** | 40 | 17 | 40 | | 15 | 36 | 18 | 30 | 21 | 32 | 20 | 36 | 19 | 40 | NA | 38 | | NA | 40 | NA | 45 | NA | 34 | NA |
| **SOFA** | 4 | 0 | 3 | | 0 | 5 | 2 | 5 | 7 | 6 | 3 | 4 | 2 | 3 | ? | 4 | | 14 | 7 | ?? | 4 | 12 | 7 | 12 |
| **SpO2 (%) *** | 83 | 96 | 86 | | 95 | 87 | 93 | 83 | 92 | 89 | 93 | 89 | 91 | 71 | NA | 90 | | 95 | 85 | 94 | 87 | 87 | 83 | 86 |
| **Assisted Ventilation** | NIV | Free | NIV | | Free | NIV | NIV | NIV | NIV | NIV | NIV | NIV | NIV | NIV | IMV | IMV | | IMV | NIV | IMV | NIV | IMV | IMV | IMV |
| **PaO_2_/FiO_2_ **** | 100 | 347 | 114 | | 347 | 86 | 325 | 95 | 316 | 103 | 328 | 81 | 376 | 50 | NA | 75 | | NA | 132 | NA | 52 | NA | 107 | NA |
| **ARDS score** | S | | | M | | S | | S | | M | | S | | S | | | S | | M | | S | | M | |
| **Total ICU admission (day)** | 5 | | | 10 | | 10 | | 29 | | 9 | | 6 | | 15 | | | 24 | | 12 | | 9 | | 14 | |
| **ICU admission to 1st infusion** | 3 | | | 8 | | 3 | | 11 | | 4 | | 1 | | 11 | | | 5 | | 5 | | 3 | | 9 | |
| **1st infusion to ICU discharge** | 2 | | | 2 | | 7 | | 18 | | 5 | | 5 | | NA | | | NA | | NA | | NA | | NA | |
| **1st infusion to death** | NA | | | NA | | NA | | NA | | NA | | NA | | 4 | | | 19 | | 7 | | 6 | | 6 | |

*: All of the patients had SpO2 less than 87 without O2 support on ICU admission day. These numbers are the average SpO2 while the patients were under positive oxygen pressure.

**: The numbers in the first column for each patient is the SpO_2_/FiO on 1^st^ day of infusion and the second column is on discharge day from ICU.

***: This patient was a known case of cardiomyopathy and diabetes which developed acute renal failure during the ICU course but he was alive at 60-days follow up and transferred to the nephrology ward. The mild dyspnea was related to the pleural effusion developed in the setting of uremia. The infusions completed within 10 days because of intermittent hemodialysis.

****: This patient showed a transient relief of dyspnea and SpO2 improvement 24-48 hours after the first injection but developed convulsion, become apneic then intubated and did not receive the third dose.

*****: This patient also showed an improvement in SpO2 and was stable on intermittent nasal O2, but developed sudden cardiac arrest.

Abbreviations: A: after cell injection, B: before cell injection, N: not applicable, D: death, ICU: Intensive care unit, M: Moderate, S: Severe, NIV: non-invasive ventilation, IMV: Invasive mechanical ventilation, SOFA: Sequential organ failure assessment.
