# Supplementary material for: Mesenchymal stem cells derived from perinatal tissues for treatment of critically ill COVID-19-induced ARDS patients: a case series
Source: Stem Cell Res Ther. 2021 Jan 29;12:91. doi: 10.1186/s13287-021-02165-4 (PMC7844804; doi:10.1186/s13287-021-02165-4)
Supplement: Supplementary file 4 — Additional file 4: Table S2. Laboratory findings before the first and after the last cell infusions. [file 13287_2021_2165_MOESM4_ESM.docx]

**Supplementary Table 2. Laboratory findings before the first and after the last cell infusions.**

|  | **Survivors** | | | | | | | | | | | | **Non-survivors** | | | | | | | | | |
| --- | --- | --- | --- | --- | --- | --- | --- | --- | --- | --- | --- | --- | --- | --- | --- | --- | --- | --- | --- | --- | --- | --- |
| **Patient #** | **2** | | **3** | | **4** | | **8** | | **10** | | **11** | | **1** | | **5** | | **6** | | **7** | | **9** | |
| **Before (B) After (A)** | **B** | **A** | **B** | **A** | **B** | **A** | **B** | **A** | **B** | **A** | **B** | **A** | **B** | **A** | **B** | **A** | **B** | **A** | **B** | **A** | **B** | **A** |
| **WBC**  **counts (× 10^3^/µl)** | 4.7 | 4.8 | 6.8 | 5.8 | 11.9 | 23 | 8.3 | 9.0 | 5.6 | 13.7 | 7.7 | 12.5 | 15 | 10 | 9.3 | 6.9 | 9.7 | 11.3 | 22.7 | 20.9 | 17.8 | 18.8 |
| **Lymphocytes (%)** | 9 | 16.5 | 14 | 18.4 | 68 | 67 | 10 | 9 | 14 | 13 | 12 | 6.4 | 2.1 | 3 | 5.3 | 9.8 | 9.7 | 2.6 | 4 | 3 | 6.7 | 6.7 |
| **Platelets (×10^6^/ µl)** | 164 | 271 | 240 | 237 | 262 | 254 | 118 | 128 | 262 | 204 | 214 | 360 | 272 | 124 | 144 | 173 | 251 | 150 | 120 | 35 | 199 | 179 |
| **PT** | 11.9 | 12 | 14 | 11 | 15 | 17 | 23 | 13 | 14.6 | 15.2 | 13.1 | 13.9 | 15.8 | 16.5 | 15.3 | 14.9 | 14.2 | 14.1 | 14.4 | 15 | 14.4 | 14.3 |
| **PTT** | 22 | 22 | 21 | 25 | 68 | 66 | 31 | 24 | 46 | 54 | 43 | 35 | 22 | 20 | 46 | 53 | 39 | 47 | 24 | 27 | 44 | 35 |
| **Hemoglobin (g/L)** | 13.3 | 13.5 | 10.6 | 11.4 | 12.4 | 9.2 | 11.8 | 10.2 | 11.8 | 11.8 | 18.6 | 16.5 | 10.5 | 11.6 | 13.6 | 8.5 | 14.2 | 12.7 | 12.8 | 10 | 8.6 | 9.3 |
| **Potassium (mmol/L)** | 4.2 | 5.1 | 3.7 | 4.1 | 3.9 | 4.2 | 3.5 | 5.2 | 4.1 | 4.6 | 4.9 | 4.8 | 5.7 | 4.5 | 4.2 | 5.1 | 5.7 | 4.5 | 4.8 | 5.1 | 4.8 | 4.9 |
| **Sodium (mmol/L)** | 136 | 138 | 139 | 142 | 138 | 138 | 131 | 133 | 137 | 137 | 138 | 143 | 140 | 141 | 150 | 138 | 140 | 141 | 140 | 143 | 138 | 142 |
| **BUN** | 18 | 33 | 10 | 11 | 37 | 29 | 27 | 69.7 | 30 | 34 | 38 | 54 | 21 | 27 | 36 | 121 | 68 | 55 | 14 | 54 | 258 | 180 |
| **Cr** | 0.9 | 1.06 | 0.62 | 0.84 | 0.9 | 0.9 | 1.26 | 7.93 | 1 | 0.9 | 1.4 | 1.1 | 0.75 | 0.92 | 1.3 | 1.9 | 1.2 | 1.1 | 1.02 | 3.2 | 3.5 | 2.6 |
| **ALT (U/L)** | 29 | NA | NA | 13 | 32 | 27 | 4200 | 139 | 30 | 70 | 33 | 134 | 25 | NA | 28 | 32 | 56 | 52 | 42 | 58 | 40 | 24 |
| **AST (U/L)** | 50 | NA | NA | 27 | 42 | 39 | 11200 | 122 | 36 | 57 | 49 | 56 | 52 | NA | 41 | 54 | 60 | 50 | 74 | 62 | 56 | 41 |
| **LDH (U/L)** | 1418 | NA | 1322 | 667 | NA | NA | 7937 | 627 | 948 | 740 | 1416 | 792 | NA | NA | 1061 | 827 | 741 | 894 | 1845 | 1819 | 842 | 912 |

NA: Not available, AST: Aspartate aminotransferase, WBC: White blood cell, RBC: Red blood cell, Cr: Creatinine, BUN: Blood urea nitrogen, ALT: Alanine aminotransferase
